# *Panax ginseng* for Frailty-Related Disorders: A Review

**DOI:** 10.3389/fnut.2018.00140

**Published:** 2019-01-17

**Authors:** Keiko Ogawa-Ochiai, Kanji Kawasaki

**Affiliations:** Department of Japanese-Traditional (Kampo) Medicine, Kanazawa University Hospital, Kanazawa, Japan

**Keywords:** *Panax ginseng*, frailty, Kampo medicine, fatigue, immune function, glucose metabolism, physical performance

## Abstract

This review aims to understand the clinical efficacy of *Panax ginseng* (PG) for managing frailty-related disorders by reviewing meta-analyses, systematic reviews, and randomized clinical trial data. PG is widely used in traditional medicine, mainly in East Asia. It has traditionally been indicated for the collapse of qi or for abandoned conditions that manifest as shallow breathing, shortness of breath, cold limbs, profuse sweating, a low pulse rate, or weakness. In accordance with these indications, PG is used for managing conditions such as aging, inflammation, and cancer. PG is also used in some functional foods or supplements. Some studies have shown the effects of ginsenosides, which are the major constituents of PG. With regard to pharmacological activities of ginseng saponins, it has been presumed that these ginsenosides are metabolized into active forms by human intestinal microbiota after being taken orally. Therefore, we focused on reviewing the data of clinical studies on PG. Although there has been no study that directly investigated the effect of PG on frailty, a number of clinical studies have been conducted to investigate the efficacy and safety of PG and its interactions with other modern ginseng medications and ginseng-containing formulas. We searched the randomized controlled trial data from 1995 to 2018 and reviewed the potential effects of PG on frailty-related disorders. We reviewed the effects of PG on glucose metabolism, fatigue, hypertension, cardiovascular disorders, chronic obstructive pulmonary disease, renal function, cognitive function, and immune function. Our review showed some evidence for the usefulness of ginseng, which suggests that it has the potential to be used for the management of aging-related and frailty symptoms, such as fatigue and hypertension. The main limitation of this review is that no study has directly investigated the effect of PG on frailty. Instead we investigated frailty-related disorders, and the limitations of the available studies were small sample sizes and a poor methodological quality; besides, only a few studies targeted elderly people, and few included placebo controls. Larger, well-designed studies are needed to determine the effect of PG on frailty in the future.

## Introduction

**Figure 1:**
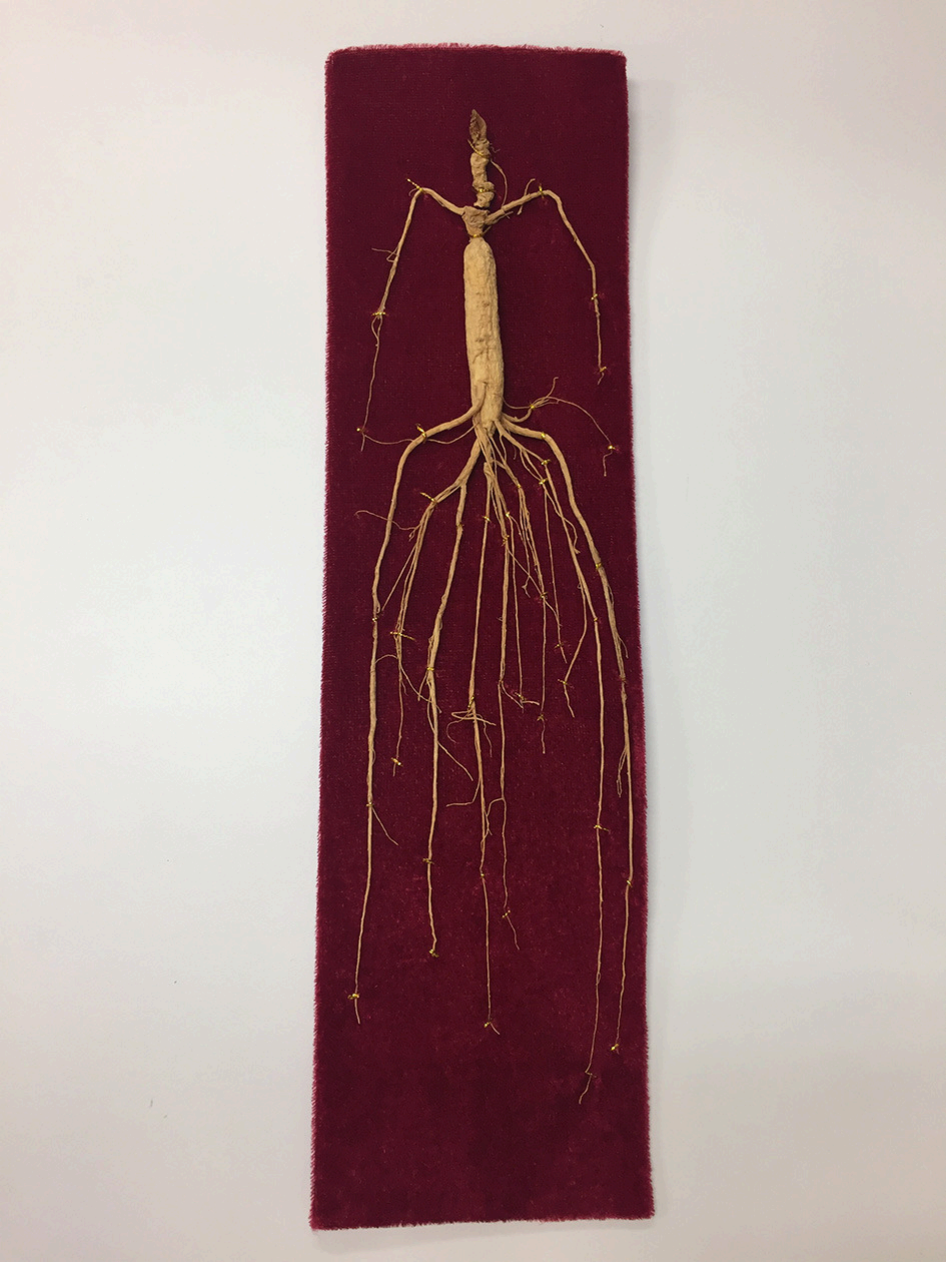
*Panax ginseng*.

*Panax ginseng* C. A. Meyer (PG) is a widely used herb from the Araliaceae family. It is commonly known as Asian or Korean ginseng. The roots of the plant are used in traditional medicine, mainly in East Asia. “Panax” means “cure-all” in Greek. The herbal root is named “ginseng” because it is shaped as a man and “Gin” means “man” in Chinese and Japanese (Figure 1).

PG has traditionally been indicated for an extreme collapse of qi or abandoned conditions such as shallow breathing, shortness of breath, coldness of limbs, profuse sweating, or weakness. PG significantly tonifies the primal qi and qi of all organs, especially that of the lungs and spleen (1). Therefore, it is used for various indications, such as aging, inflammation, stress, or cancer, and also used in some functional foods or supplements.

PG was originally dried in the sun before use; however, a steaming method has been developed to facilitate PG digestion. PG is harvested after 4–6 years of growth and is classified into two types, depending on the processing method, white ginseng (harvested after 4–6 years of growth and dried after peeling) and red ginseng or Korean Red Ginseng (KRG; harvested at 6 years, steamed, and dried).

The therapeutic benefits of PG are well-established and are attributable to a unique mix of triterpenoid saponins (ginsenosides). Shibata et al. (2) first identified the structures of various ginsenosides. Regarding the pharmacological activities of ginseng saponins, it is presumed that these ginsenosides are metabolized into active forms by human intestinal microbiota after being taken orally.

Frailty encompasses a range of physical, mental, and social problems, such as physical disability, cognitive dysfunction, depression, and economic difficulties (3). It is defined as a clinical syndrome in which three or more of the following conditions occur: unintentional weight loss (10 lb in a previous year), self-reported exhaustion, weakness (loss of grip strength), reduction in the walking speed, and low physical activity. Among these symptoms, weakness and slowness are related to sarcopenia, which suggests that sarcopenia is the main underlying cause of physical frailty.

**Figure 2:**
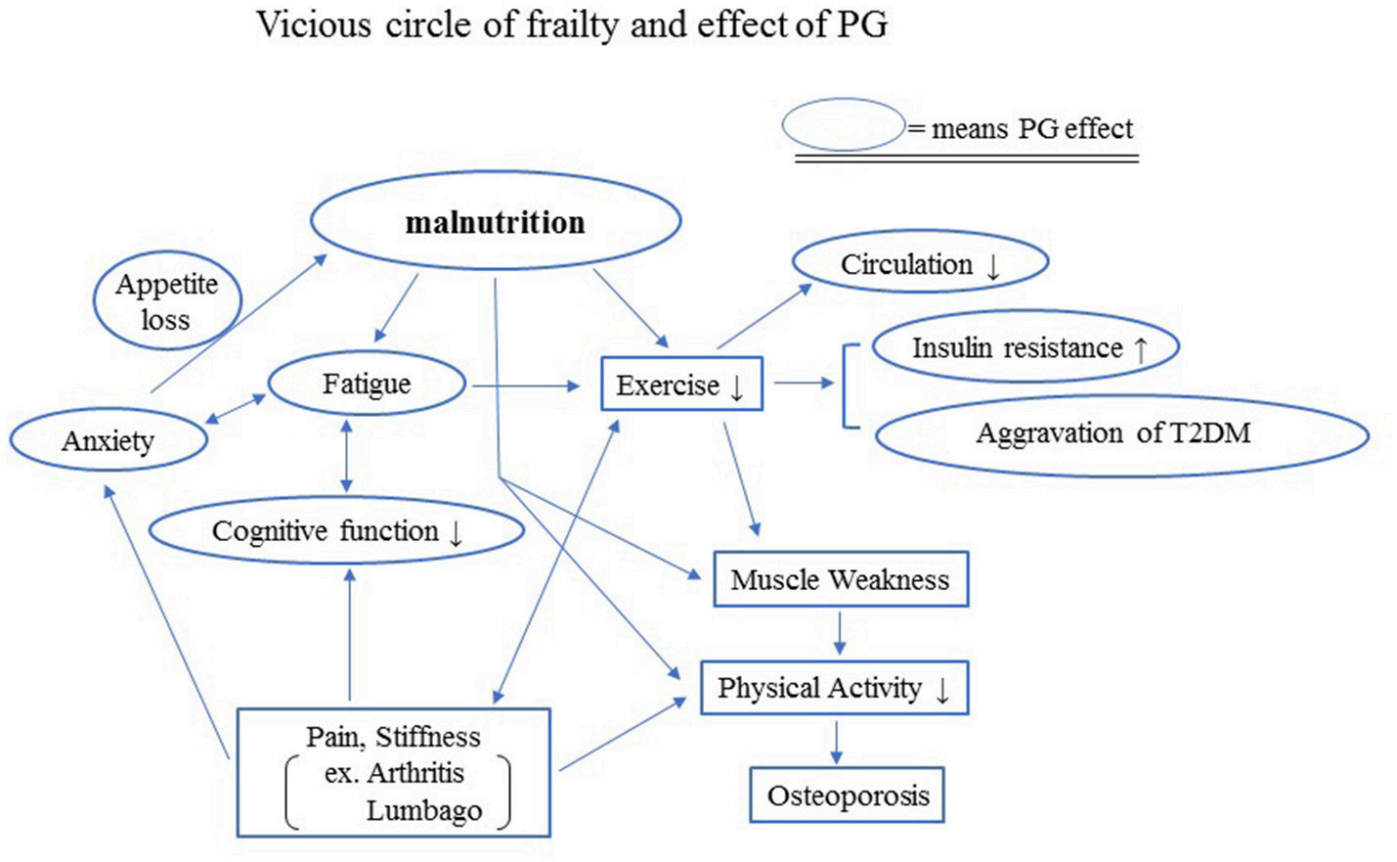
Vicious circle of frailty and effects of PG. Exercise and nutritional care are critical for the prevention of frailty. Elderly people often lose appetite, which causes malnutrition. Malnutrition makes it difficult for the elderly to perform exercise therapy because of the weakness, fatigue, declining cognitive function, and various physical symptoms, including pain, although the most effective non-drug therapy for the management of pain disorders is exercise. The lack of sufficient exercise increases insulin resistance and may aggravate T2DM.

Exercise and nutritional care are critical for the prevention of frailty. Elderly people often find it difficult to perform exercise therapy because of weakness and various physical symptoms, including pain. Although pain control plays an important role in managing frailty, non-steroidal anti-inflammatory drugs, anticonvulsants, or opioids are sometimes not suitable for the use in elderly patients because the drugs may cause side effects such as gastrointestinal disturbance, renal dysfunction, or delirium. Ironically, the most effective non-drug therapy for the management of pain disorders is exercise. Thus, a vicious cycle of frailty is likely to occur in the elderly (Figure 2).

In this review, we aimed to evaluate the effectiveness and safety of oral administration of PG for the management of frailty and aging-related symptoms by reviewing relevant meta-analyses, reports of randomized controlled trials (RCTs), and systematic reviews of frailty-related disorders. A decrease in the exercise capacity and motivation and a decline in endocrine and cognitive functions are thought to exacerbate frailty. Therefore, in this article we reviewed the effects of PG on glucose metabolism, fatigue, hypertension, cardiovascular disorders, chronic obstructive pulmonary disease (COPD), and renal, cognitive, and immune functions.

## Methods

**Figure 3:**
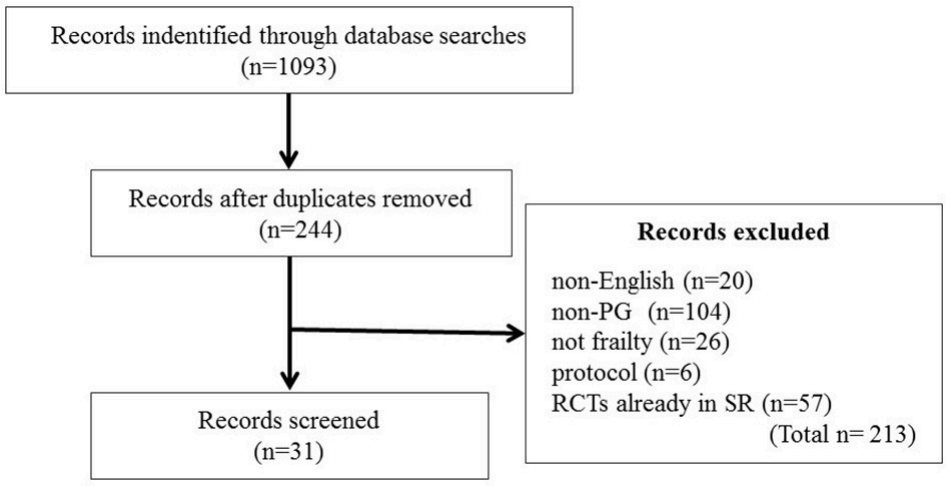
Flow diagram of the literature search. RCT, randomized controlled trial.

Many clinical studies have been conducted to investigate the PG efficacy, safety, and interaction with other modern ginseng medications and ginseng-containing formulas. We searched the PubMed, Ovid Technologies, and Cochrane Library databases for clinical controlled trials, RCTs, meta-analyses, and systematic reviews published from 1995 to February 2018 to review the effect of PG on frailty. The following search terms were used, without language restrictions: “ginseng” AND (“randomized controlled trial” OR “RCT” OR “clinical controlled trial” OR “control trial” OR “systematic review” OR “meta-analysis” OR “double-blind” OR “single-blind” OR “clinical trial”). Studies in which PG was administered orally were included without considering the patients' sex or underlying disease. We did not limit the inclusion based on the types of control groups. Literature on ginseng species other than PG was excluded from this review (Figure 3). The two authors (KO-O and KK) independently screened the eligible studies and discussed discrepancies until an agreement was reached. Studies that indirectly investigated the effects of PG on frailty, glucose metabolism, fatigue, hypertension, cardiovascular disorders, COPD, renal function, cognitive function, and immune function were selected.

## Results

No study has directly investigated the effect of PG on frailty, so we investigated the effect of PG on frailty-related disorders.

### Glucose Metabolism

In a systematic review focused only on PG (4), a definitive conclusion has not been reached because few studies were included and the treatment regimens varied. Therefore, future studies with an adequate sample size and power, standardized treatment regimens, and a rigorous methodology are needed.

### Fatigue

Bach et al. (5) conducted a meta-analysis of 12 RCTs to investigate the efficacy of ginseng supplements in alleviating fatigue. RCTs that investigated the efficacy of ginseng supplements in fatigue reduction and enhancement of physical performance in comparison with that of placebo were included, of which nine investigated PG. The meta-analysis showed no significant association between ginseng supplementation and the enhancement of physical performance because few relevant RCTs were published and the sample size was small across RCTs. The aforementioned RCTs were conducted in healthy subjects, but some positive results suggested an anti-fatigue effect of PG in patients with idiopathic chronic fatigue (ICF) and chronic fatigue syndrome (CFS).

KRG (harvested at the age of 6 years, steamed, and dried) was assessed for its anti-fatigue effects in patients with non-alcoholic fatty liver disease (NAFLD). Eighty patients with NAFLD were randomized to receive KRG or placebo for 3 weeks, in addition to counseling on healthy eating and regular exercise. Liver function, proinflammatory cytokines, adiponectin, antioxidant activity, and a fatigue score were measured and compared, according to the body mass index, between the KRG and placebo groups. The results showed that KRG might be effective in reducing proinflammatory cytokine levels and fatigue in overweight patients with NAFLD. The mean level of tumor necrosis factor-α significantly decreased in the KRG group after treatment, compared with that at baseline, and there was a significant increase in the changes in adiponectin levels in the KRG than in placebo groups. In overweight patients, the fatigue score significantly decreased in the KRG group (6).

### Hypertension and Cardiovascular Diseases

A systematic review has provided evidence for the efficacy of KRG in reducing blood pressure in patients with prehypertension and acute and long-term hypertension (7). However, the interpretation of the findings of the meta-analysis was limited by the inclusion of low-quality RCTs in the study design. Another systematic review examined the evidence for the efficacy of ginseng (*Panax* spp.) in the management of cardiovascular risk factors, including high blood pressure, an abnormal lipid profile, and a high blood glucose level, and summarized reported cardiovascular adverse events. Some included studies suggested that the ginseng use caused a small reduction in the blood pressure (range: 0–4%); however, the evidence provided did not support the use of ginseng for managing cardiovascular risk factors, partly because the majority of the studies included were short-term studies (8).

A meta-analysis of 18 eligible RCTs provided moderate evidence that ginseng-based medicines were more effective than nitrates for treating angina pectoris, at a significant overall odds ratio of 3.00 (*P* < 0.00001) for symptomatic improvement and 1.61 (*P* = 0.001) for electrocardiographic improvement. However, there were limitations for generalization because of a short follow-up period (9). A systematic review on the use of the PG-containing Shexiang Baoxin Pill for ischemic cardiovascular diseases showed that the addition of the pill to conventional treatments may have beneficial effects on long-term outcomes of non-ST elevation acute coronary syndromes, without serious adverse events (10).

### Chronic Obstructive Pulmonary Disease

A systematic review has shown improvements in the quality of life (QoL) and lung function, based on the changes in forced expiratory volume in 1 s (FEV_1_) and FEV_1_ % predicted between a ginseng-treated group and placebo-treated group, no treatment control, and non-ginseng formula-treated group [(11)]. Another review has shown that PG may improve respiratory muscle strength and lung function (12). Further, a systematic review that evaluated an oral Chinese herbal medicine combined with pharmacotherapy for stable COPD showed clinically meaningful benefits in terms of an improved body mass index, airflow obstruction, dyspnea, and exercise capacity (BODE) index and improved results of a 6-min walk test. In the studies included in the review, PG was one of the most used crude drugs in the formulas (13).

### Renal Function

Recent studies have shown that ginsenosides can be used to treat early chronic kidney disease (14), and an RCT showed that ginsenoside Rb1 ameliorated the renal function in patients with early chronic kidney disease. Compared with those in the placebo group, renal function parameters (creatinine and urea clearance), oxidative stress, and inflammation were significantly reduced in the ginsenoside-treated patients (14).

### Cognitive Function

Reay et al. (15, 16) have reported anti-mental fatigue effects of PG, indicated by the improvements in cognitive performance of healthy volunteers in serial clinical studies. Other researchers have also demonstrated a positive effect of PG on the memory of healthy volunteers [(17), (18)].

Lee et al. (7) have evaluated the effectiveness and safety of ginseng for Alzheimer's disease (AD) patients based on the data from four RCTs involving 259 participants. The review showed that the effects of ginseng on AD were inconclusive, and another review has made similar conclusions [(19)]. However, one study found that the results favored ginseng treatment, as indicated by AD assessment scale-cognitive (ADAS-cog) subscale scores at 24 weeks.

Thus, it has not been proven that PG is effective for AD; however, there is no curative treatment for AD. Although the effectiveness of PG for AD is unclear, it has been empirically and widely used in the treatment of AD in Asian countries. Recently, many studies have reported the potential effects of some PG-containing formulas on cognitive function. Kudouh et al. (20) evaluated the long-term effects of a traditional Japanese Kampo medicine, ninjin'yoeito (NYT), on cognitive impairment and mood status in patients with AD over a 2-year period. The researchers found significant improvements in the Japanese version of the ADAS-cog scale scores and in the Neuropsychiatric Inventory depression scores of patients who received combination therapy with donepezil and NYT. A 2-year follow-up of the patients receiving the combination therapy showed an improved cognitive outcome and alleviation of AD-related depression.

### Immune Function

It has been reported that ginsenoside Rg1 stimulated the proliferation of lymphocytes. Other studies have shown that ginsenosides may enhance the cellular immune function (21).

A meta-analysis that evaluated the effects of ginseng consumption on cancer risks has identified nine studies (five cohort studies, three case-control studies, and one RCT), involving 7,436 cases and 334,544 participants. The findings of this meta-analysis indicated that ginseng consumption was associated with a significantly decreased risk of cancer. The summary relative risks for ginseng intake vs. no ginseng consumption were 0.77 for lung cancer, 0.83 for gastric cancer, 0.81 for liver cancer, and 0.77 for colorectal cancer. Thus, clinical trials of PG are warranted to determine whether it can prevent cancer (22).

### Safety

A systematic review has found that PG has a very safe profile and is rarely associated with adverse events or drug interactions. The few effects reported were mild and transient, such as insomnia, headache, a skin disorder, nasal bleeding, or hot flashes (23).

A survey of published literature has identified 16 articles involving the use of herbal remedies in the elderly population. Remedies containing PG are frequently used by the elderly; however, not all interactions and side effects have been elucidated. It is therefore incumbent that medical doctors are aware of the use of herbal products, including PG, by the elderly when prescribing a specific pharmacological treatment. The lack of communication between medical professionals and patients about the use of herbal supplements may pose a problem (24).

PG has been reported to interact with phenelzine, a monoamine oxidase inhibitor, and the interaction may cause sleeplessness, tremors, and headache (25). Three studies investigated the interaction between ginseng and warfarin, and the results differed depending on the type of ginseng used. PG had little or no influence on coagulation (26). A review of herbal medicinal products (HMPs) used by adults aged >65 years old has reported that the most frequently used HMPs in the US were those containing *Ginkgo biloba*, garlic, ginseng, St. John's wort, echinacea, saw palmetto, evening primrose oil, and ginger. The prevalence of concurrent use of prescription drugs and HMPs is substantial, ranging from 5.3 to 88.3% among adults aged >65 years old, and potential interactions have been reported. The most frequently reported effect of herb–drug interactions is bleeding due to the use of *G. biloba*, garlic, or ginseng with aspirin or warfarin. Knowledge of the extent and manner in which older adults combine prescription drugs with HMPs will aid healthcare professionals in appropriately identifying and managing patients at risk. Comprehensive online databases such as Micromedex, Natural Medicines, and Stockley's Drug Interactions can be useful (27).

Phytochemical-mediated modulation of cytochrome P-450 (CYP) activity may underlie many herb–drug interactions. Some evidence suggests that CYP activity may decrease in the elderly; therefore, clinical investigations need to be focused on this population. It has been reported (28) that the inhibition of CYP2D6 by PG was statistically significant, but the magnitude of the effect (~7%) did not appear clinically relevant. Meanwhile, PG did not affect the CYP1A2 activity.

## Discussion

In this review, we evaluated the clinical efficacy of PG for frailty by reviewing RCT data, meta-analyses, and systematic reviews of the effect of PG on frailty-related disorders. Although no study has directly investigated the effect of PG on frailty, we were able to obtain knowledge on diseases leading to prevention and treatment of frailty.

Healthy life year's expectancy (HLYE) is determined by the interaction of psychophysical and socioeconomic factors during an individual's life course. Considering from a global point of view, the HLYE is deeply involved with frailty. Although the life expectancy at birth has increased over the past decade, HLYE has not increased over the same period. Prevention is the likely key to increasing HLYE. For example, ~50% of reduction in mortality from cardiovascular diseases is the result of prevention-based activities, such as a change in lifestyle or smoking cessation. With regard to prevention, frailty is an important factor for understanding the condition of older patients. Unfortunately, prevention programs targeting older adults are limited because frailty limits activities of these patients, thus resulting in a vicious cycle (Figure 2).

The World Health Organization estimates that more than 180 million people worldwide suffer from diabetes, with 90% of the patients suffering from T2DM. Serious long-term complications of diabetes greatly increase frailty. Diabetes was understood as yin deficiency in traditional East Asian medicine. According to *Shang Han Za Bing Lun* (*Treatise on Cold-Induced and Miscellaneous Diseases*), PG could be used to treat thirst, probably caused by DM. Thus, PG has long been used in the treatment of diabetes. Although clinical reviews could not prove the effect of PG on DM, two systematic reviews of ginseng other than PG showed favorable effects in glucose metabolism. One systematic review showed a modest yet significant improvement in fasting blood glucose levels in people with and without diabetes after the administration of ginseng (29). Another systematic review of ginseng-related therapies in type 2 diabetes mellitus (T2DM)T2DM (30) showed the benefit of ginseng supplementation in improving glucose control and insulin sensitivity in patients with T2DM or impaired glucose intolerance. And the effects of PG or its components on blood glucose control have been well-documented in experimental models. Ginsenosides have been shown to activate the AMP-activated protein kinase (AMPK) pathway (31), and activation of the AMPK pathway has been proposed as a mechanism for the suppression of hepatic gluconeogenesis and steatosis (32). Therefore, it has been suggested that ginsenosides may decrease the ATP biosynthesis and lower the blood glucose level. DPG-3-2, a component of Ginseng Radix, was shown to lower the blood glucose level and to stimulate the insulin release and biosynthesis in diabetic animals and in pancreas preparations from animals with hyperglycemia (33).

Some results suggest anti-fatigue effects of PG in patients with ICF and CFS. According to an RCT, the fatigue score significantly decreased in the KRG group of overweight patients. KRG also significantly improved the liver function test results, which indicates that KRG may improve the physical and mental fatigue. Most of the studies reviewed were not in elderly people, but it may be possible to presume that PG is also effective for elderly people. It is expected that RCTs involving elderly people will be conducted.

PG has also been reported to reduce blood pressure. Combination treatments for blood stasis are considered more effective for the management of cardiovascular diseases. However, the use of ginseng for managing cardiovascular risk factors was not supported by evidence, partly because the majority of the studies included were short-term studies, while cardiovascular risk must be observed for a long time. It has been shown in animal models that ginsenosides had protective effects on myocardial ischemia/reperfusion injury by mediating the activation of the phosphoinositide 3-kinase pathway and phosphorylation of protein kinase B (34). It has also been reported that ginsenoside Rb3 inhibited the angiotensin II-induced proliferation of vascular smooth muscle cells (35).

Regarding COPD, characterized by progressive, non-reversible airflow limitation, PG may improve respiratory muscle strength, lung function, and the BODE index. In Kampo medicine, stable COPD represents a pattern of qi deficiency, involving either the lung or spleen as described in the Introduction (1). PG has an advantage for symptom management, QoL improvement, and reduction of exacerbation because current pharmacotherapy does not prevent the progression of COPD, nor does it improve the lung function.

Renal function decreases with aging and is thus one of the most important factors in frailty. It has been shown that ginsenosides can be used to treat early chronic kidney disease. This disease is progressive, and thus long-term observation would be needed for elderly people to investigate the effect of PG on renal function.

With regard to cognitive function, AD and other dementias are a global health challenge and are remarkable in terms of the prevalence, costs, and impact. In 2010, an estimated 35.6 million people were reported to have AD and other dementias worldwide, and the number is predicted to reach 66 million by 2030 and 115 million by 2050. In this review, the effectiveness of PG for AD was found to be unclear, but PG has been empirically and widely used in the treatment of AD in Asian countries. NYT was reported to be effective for cognitive impairment and mood status in patients with AD over a 2-year period. Although the study was performed using a Kampo formula extract, the results might have been due to the medicinal efficacy of PG, which is the major component in this formula. The Kampo formula might be more effective than PG alone. Beneficial effects of PG in an animal model of AD included a significant improvement of behavioral profiles in rats with advanced glycation end product (AGE)-induced AD. PG also significantly reduced the malondialdehyde level, increased the glutathione content, and increased the superoxide dismutase activity in the hippocampus. Moreover, PG significantly decreased the expression of the receptor for AGEs (RAGE) and nuclear factor-κB. The blockade with an anti-RAGE antibody could significantly reduce the AGE-induced impairments and regulate the expression of these proteins (36). It has also been reported that ginsenosides could significantly improve behavioral profiles and repair the damage to the hippocampus by reducing the phosphorylation of the amyloid-β peptide and tau protein. Ginsenosides could also increase the γ-aminobutyric acid, acetylcholine, and dopamine levels and decrease those of glutamate and aspartic acid in the hippocampus and cortex and increase glycine and serotonin levels in the blood (37).

PG is widely used as an adjuvant in traditional medicine to enhance human immunity. Hundreds of studies conducted *in vitro* and in animal models have reported the anticancer or chemopreventive effects of PG. The anticancer effects of ginseng are mainly associated with the improvements in cell-mediated immunity, including activation of cytotoxic T cells and natural killer cells, while other mechanisms, such as oxidative stress, apoptosis, and angiogenesis, are also involved. However, the clinical effects of PG on cancer prevention are not clear. There are relatively few clinical studies focused on the immunomodulatory properties of PG.

Many elderly patients use herbal formulas for the relief of aging-related symptoms, which are not easily treated by conventional medicine. These patients usually have a number of chronic diseases and take more prescribed medications than the younger population does.

In our review, it was found that PG had a safe profile and was rarely associated with serious adverse events. Few reported effects included mild symptoms such as insomnia, headache, skin disorders, nasal bleeding, or hot flashes. Regarding drug interactions, PG inhibition of CYP2D6 was statistically significant, but the magnitude of the effect did not appear clinically relevant. PG can be used relatively safely in the elderly, but careful observation is necessary.

Many studies have not specified which PG they used; meanwhile, it is difficult to determine the efficacy of herbal crude drugs, unless the method of processing is clear. The *Divine Husbandman's Classic of the Materia Medica*, the earliest existing monograph of traditional Chinese medicine, prepared ~4,000 years ago, mentions that PG governs the tonification of five organs, quiets the consciousness, settles the ethereal soul and the corporeal soul, arrests palpitations and anxiety, expels pathogenic qi, brightens the eyes, opens the heart, and strengthens the resolve. According to this book, the use of ginseng can delay aging.

Accumulating evidence strongly suggests that ginsenosides are prodrugs that are activated in the body upon deglycosylation by intestinal bacteria and esterification with fatty acids (38). Orally ingested ginsenosides pass through the stomach and small intestine, without being broken down by either the gastric juice or liver enzymes, into the large intestine, where ginsenosides are deglycosylated by colonic bacteria, followed by their transit to the circulation. Colonic bacteria cleave the oligosaccharide connected to the aglycone in a stepwise manner, from the terminal sugar, to afford the major metabolites, 20*S*-protopanaxadiol 20-*O*-β-D-glucopyranoside and 20*S*-protopanaxatriol. These metabolites are further esterified with fatty acids. The resultant fatty acid conjugates are still active molecules, which are sustained longer in the body than are the parental metabolites. Changes in the microbiota with the age may influence the effect of PG. Therefore, further investigations of clinical use of PG in elderly patients are necessary.

Fermented ginseng products, containing ginsenoside metabolites, may have merit for standardizing ginseng efficacy (38). Therefore, it is necessary to distinguish ginseng products according to their spices and the method of processing. Previous reports have suggested further possibilities for PG application. We hope that further studies will elucidate the potential of PG for application in an aging society. The efficacy and safety of PG need to be scientifically assessed by verified observations.

Japan is an advanced aging society. Because most Kampo formulas are covered by the national insurance system, they have already been used as superior medicines for the management of frailty (39). Although this latter study was conducted on a Kampo formula extract, NYT, the results may reflect the medicinal efficacy of PG, which is the major crude drug in this formula. It has been empirically predicted that the Kampo prescription is more effective than PG alone. Among Kampo formulas, rikkunshito, hochuekkito, minjinyoeito, and kamikihito contain PG, and these formulas have been used for a long time to ameliorate malnutrition or a loss of physical activity. They may also be effective to prevent frailty.

The main limitation of this review is that no study has directly investigated the effect of PG on frailty. Instead we investigated frailty-related disorders, and the limitations of the available studies were small sample sizes and a poor methodological quality; besides, only a few studies targeted elderly people, and few included placebo controls. Larger, well-designed studies are needed to determine the effect of PG on frailty in the future.

## Author Contributions

KO-O conceived and designed the study and developed the hypothesis. KO-O and KK analyzed the data. KO-O wrote the manuscript. Both authors discussed the results and contributed to the final manuscript.

### Conflict of Interest Statement

The authors declare that the research was conducted in the absence of any commercial or financial relationships that could be construed as a potential conflict of interest.
